# Responses of Gut Microbial Community Composition and Function of the Freshwater Gastropod *Bellamya aeruginosa* to Cyanobacterial Bloom

**DOI:** 10.3389/fmicb.2022.906278

**Published:** 2022-05-13

**Authors:** Tianying Lyu, Jinyong Zhu, Xianming Yang, Wen Yang, Zhongming Zheng

**Affiliations:** School of Marine Science, Ningbo University, Ningbo, China

**Keywords:** *Bellamya aeruginosa*, gut microbiota, cyanobacterial bloom, microcystins, PICRUSt2

## Abstract

Freshwater gastropods are widely distributed and play an important role in aquatic ecosystems. Symbiotic microorganisms represented by gut microbes can affect the physiological and biochemical activities of their hosts. However, few studies have investigated the response of the gut microbial community of snails to environmental stress. In this study, the dynamics of the gut microbiota of the gastropod *Bellamya aeruginosa* were tracked to explore their responses in terms of their composition and function to cyanobacterial bloom. Differences in gut microbial community structures during periods of non-cyanobacterial bloom and cyanobacterial bloom were determined. Results showed that the alpha diversity of the gut microbiota exposed to cyanobacterial bloom was lower than that of the gut microbiota exposed to non-cyanobacterial bloom. The main genera differentiating the two periods were *Faecalibacterium, Subdoligranulum, Ralstonia*, and *Pelomonas*. Microcystins (MCs) and water temperature (WT) were the primary factors influencing the gut microbial community of *B. aeruginosa*; between them, the influence of MCs was greater than that of WT. Fourteen pathways (level 2) were notably different between the two periods. The pathways of carbohydrate metabolism, immune system, environmental adaptation, and xenobiotics biodegradation and metabolism in these differential pathways exhibited a strong linear regression relationship with MCs and WT. Changes in the functions of the gut microbiota may help *B. aeruginosa* meet its immunity and energy needs during cyanobacterial bloom stress. These results provide key information for understanding the response pattern of freshwater snail intestinal flora to cyanobacterial blooms and reveal the underlying environmental adaptation mechanism of gastropods from the perspective of intestinal flora.

## Introduction

With the sustained intensification of anthropogenic activities, global climate warming and eutrophication have provoked the widespread occurrence of cyanobacterial blooms in various water bodies worldwide (Paerl, 2018). The increasing frequency, magnitude, and duration of cyanobacterial blooms not only seriously disrupt aquatic ecosystem functions but also pose a major threat to the survival of aquatic animals through the release of cyanotoxins (Huisman et al., 2018). Owing to the high spatial overlap between the cyanobacterial scum accumulation zones and the main activity areas of gastropods during cyanobacterial blooms, and because the major bloom season also coincides with the breeding season of gastropods (Lance et al., 2010), the interactions between cyanobacterial blooms and freshwater snails are inevitable.

Cyanotoxins, represented by microcystins (MCs), may accumulate in the various tissues of snails through intestinal (consumption) or gill/skin (absorption) epithelia (Massey et al., 2018), resulting in a series of adverse reactions in gastropods (Bownik, 2016). Numerous studies have investigated the response of freshwater snails to cyanobacterial blooms and their toxins from multiple layers and perspectives, including biochemistry and histopathology (Zhu et al., 2011), transcriptomics (Qiao et al., 2018a), metabolomics (Yang M. J. et al., 2022), development (Lei et al., 2017), fecundity (Qiao et al., 2018b), feeding ecology (Qiu et al., 2017), and community dynamics (Gérard and Lance, 2019) and structure (Gérard et al., 2009). However, almost all of these studies have focused on the snails themselves and have failed to consider the dynamics and functions of their symbiotic microbes when exposed to cyanobacterial blooms.

With the rapid development of high-throughput sequencing technology, we can effectively and comprehensively gain insights into the composition and dynamics of symbiotic bacteria associated with gastropods, especially gut microbiota (Hu et al., 2018; Li et al., 2019; Hao et al., 2020). Gut microbes not only provide various key digestive enzymes for the snail host (Brendelberger, 1997; Ito et al., 2019) but also strongly affect many physiological processes, such as uric acid oxidation (Van Horn et al., 2012), cold hardiness (Nicolai et al., 2015), and amino acid and energy metabolism (Grandiosa et al., 2018), which were once thought to depend only on the gastropod host genome. Some gut microbes can even adhere to intestinal mucosa and stimulate immune response that is mediated by the regulation of the expression of Toll-like receptors in snail intestinal mucosa (Dushku et al., 2019). Moreover, unlike the largely static host genome, the symbiotic microbiome is highly plastic and can rapidly respond to changes in host diet or environmental conditions through changes in community composition, mutations, exchange of genetic material with bacteria from the environment, or changes in gene expression (Shapira, 2016), as well as enhance host adaptation to the fast and drastic environmental changes by influencing various physiological activities of the host (Gilbert et al., 2015). Thus, the gut microbiota is an important source of metabolic flexibility for the host, and it might be a key, yet understudied, factor in tolerance and adaptation to environmental stress (Macke et al., 2017).

*Bellamya aeruginosa* is an indigenous freshwater snail in China. It mainly lives in the littoral zones of rivers and lakes and plays a key role in aquatic ecosystems as an important link between primary producers and secondary consumers (Gong et al., 2009). Owing to its high sensitivity to environmental toxins and strong enrichment ability of pollutants, it is considered as an ideal indicator of ecosystem health, and it has been recommended as a model organism for freshwater ecotoxicology studies (Ma et al., 2010). As far as we know, the composition and dynamic characteristics of the gut microbiota of *Bellamya* have not been described nor have their response pattern and mechanism to environmental stress been reported. Characterizing the responses of gut microbiota to cyanobacterial bloom will help us understand how changing environmental conditions can influence symbiotic microbes and reveal the mechanisms of gastropod-microbiome interactions. The aim of this study was to determine how the intestinal bacterial community and its function change during the snail host exposure to toxic cyanobacterial bloom. We investigated monthly variation in the gut microbiota of *B. aeruginosa* in a water body experiencing cyanobacterial bloom during the activity period of *Bellamya* to address this question and test the following hypotheses: (1) the composition and function of gut microbiota are expected to be notably different between periods of non-cyanobacterial bloom and cyanobacterial bloom; (2) the dynamic responses of the gut microbiota in *B. aeruginosa* are related to environmental conditions and the degree of MCs accumulation.

## Materials and Methods

### Investigation Site and Sample Collection

*B. aeruginosa* individuals were collected by hand picking from the littoral zone of a eutrophic shallow pond (ca. 0.35 ha, ca. 1.5 m deep, 29.91°N, 121.64°E) at Ningbo, eastern China. Although the pond has undergone several ecological remediations (dredging and macrophyte restoration), cyanobacteria-dominant (mainly *Microcystis* spp.) algal blooms still frequently occur. Snail samples (three individuals) and water samples were collected every month during the activity period of *Bellamya* (from March to November 2020). Then, the snail samples were individually placed in a plastic container with sterile water and transported to the laboratory. After scrubbing their surface with 70% ethanol, their shells were carefully crushed, and then their intestines and hepatopancreases were dissected for gut microbiota studies and MCs content analysis, respectively.

### Environmental Analysis

Water temperature (WT), pH, and dissolved oxygen (DO) were recorded *in situ* by using a multi-probe (YSI 6000, YSI Inc., Yellow Springs, USA). Secchi depth (SD) was estimated by a 0.2 m diameter black-white Secchi disk. Levels of nitrite (NO_2_-N), nitrate (NO_3_-N), ammonium (NH_4_-N), orthophosphate (PO_4_-P), total nitrogen (TN) and total phosphorus (TP) were analyzed following standard methods by using an automated spectrophotometer (Smart-Chem 200 Discrete Analyzer, Westco Scientific Instruments, Brookfield, USA). Concentration of chlorophyll *a* (Chl-*a*) was measured via the hot ethanol method (Chen, 2006).

### Phytoplankton Analysis

Phytoplankton sample (ca. 250 mL) was obtained from the pond each month and immediately fixed with 1% Lugol's iodine solution. Taxa were counted in sedimentation chambers (Hydro-Bios Apparatebau GmbH Kiel, Germany) by using an inverted microscope (CK2, Olympus Corporation, Tokyo, Japan) according to quantitative Utermöhl method (Utermöhl, 1958). Phytoplankton biomass was calculated volumetrically through the OptiCount software (SequentiX, Klein Raden, Germany). The specific density of phytoplankton cells was assumed to be 1 g cm^−3^ (wet weight).

### Quantification of MCs

The hepatopancreases were weighed before and after lyophilization. Microcystin extraction was conducted as previously described by Yang W. et al. (2022). MCs concentrations in the hepatopancreases were determined by the immunoassay method by using an ELISA test kit (sensitivity: 0.1 μg/L) with a detection range of 0.1–2 μg/L microcystin (Beacon Analytical Systems, Portland, ME, USA) following the manufacturers' instructions. The Beacon Microcystin Plate Kit was not available for the identification of microcystin variants. Thus, MCs concentration was expressed as the equivalent of MC-LR.

Recoveries of MCs extracted and matrix effects were determined by spiking with MC-LR standard (3 μg/g dry weight, purity > 98%; MedChemExpress, Monmouth Junction, NJ, USA) (Zhang et al., 2016). The response was compared with 100% methanol spiked with the same amount. The efficiency of MCs extraction was 91.6%. Matrix effects were negligible for the hepatopancreases of the snails (4.3% of differences between matrix and methanol results). Owing to the high recoveries of MCs extracted and the low matrix effects of the assay methods, the data on MCs contents in the present study were the actual determined data, without any additional calculation using recoveries and matrix effects.

### DNA Extraction, Amplification, and Pyrosequencing

Snail intestinal genomic DNA was extracted from individual snails following the standard DNA extraction procedure with a DNA extraction kit (MinkaGene Bacterial DNA Kit) in accordance with the manufacturer's instructions. PCR amplification of the 16S rRNA gene V3–V4 variable regions was performed using the bacterial universal primers 338 F (5′-ACTCCTACGGGAGGCAGCA-3′) and 806 R (5′-GGACTACHVGGGTWTCTAAT-3′) under the following conditions: 95°C for 3 min, followed by 28 cycles of 95°C for 30 s, 50°C for 30 s, and 72°C for 45 s; with a final extension step at 72°C for 10 min. PCR analysis was performed in triplicate for each sample. The PCR amplicons were purified and quantified with a PCR fragment purification kit (Takara, Japan) and a Quant-iT Pico Green dsDNA Quantification Kit (Invitrogen, Carlsbad, CA, USA), respectively. The equimolar PCR products from each sample were combined into equimolar ratios for paired-end library preparation and sequenced on an Illumina MiSeq Platform (Illumina, San Diego, CA, USA).

### Processing of Original Sequencing Data

Paired-end sequence reads were merged using USEARCH (version 11). The paired reads were merged into the Fastq mergepairs function and filtered with a “maxee” value of 1.0. Unique sequence reads were obtained using the fastx_uniques function, and then noise reduction was performed using the unoise3 (unoise_alpha = 4, minsize = 6) algorithm to correct errors. The remaining sequences were then screened to remove chimeras and clustered into zero-radius operational taxonomic units (ZOTUs) with 97% similarity or more, after which all quality-filtered reads were mapped to these ZOTUs (Edgar, 2016). The representative sequences for each ZOTU were assigned to taxonomic groups by using the RDP classifier within the SILVA database (16s_v138) clustered at 99% similarity.

### Data Analysis

ZOTUs with a relative abundance higher than 0.001% of all ZOTUs were included for further analysis. The gut microbes data were Hellinger transformed, and the environmental factors were normalized before analysis.

The Bray-Curtis dissimilarity of gut microbial community composition was calculated to build the matrix to identify the relationships among samples by using the function vegdist in the “vegan” package. Non-metric multidimensional scaling (NMDS) was implemented to evaluate the overall differences in gut microbial community among different months by using the function metaMDS in the “vegan” package. An analysis of similarity (ANOSIM) was performed to determine the significance of differences in gut microbial community composition between periods of non-cyanobacterial and cyanobacterial blooms by using the function anosim in the “vegan” package. Alpha diversity indices, including Shannon-Wiener index, Simpson index, Pilou's evenness, and richness, were calculated using the R package “vegan.” Student's *t*-test was used to identify significant differences in the alpha diversity indices between the two periods. The top 10 taxa in terms of abundance at the phylum and class levels were selected for creating stack maps in the “ggplot2” package. Furthermore, bacterial taxa differentially represented between the two periods at the species or higher taxonomic levels (depending on taxa annotation) were identified by performing linear discriminant analysis (LDA) coupled with effect size (LEfSe) (http://huttenhower.sph.harvard.edu/galaxy) (Segata et al., 2011). The environmental factors that have a significant effect on the gut microbial community were assessed by performing a forward selection model with Monte Carlo permutation tests by using the “packfor” package. Multicollinearity among variables was assessed by calculating variance inflation factors (VIF) by using the vif.cca function in the “vegan” package. Then, redundancy analysis (RDA) was conducted to analyze the relationship between the selected factors and the gut microbial community composition. Variance partitioning analysis (VPA) was performed to quantify the fractions of variation explained by the different forward-selected environmental variables by using the varpart function in the “vegan” package with 999 permutation test. PICRUSt2 (https://github.com/picrust/picrust2) was used to obtain information at different pathway levels (levels 1–3) compared with Kyoto Encyclopedia of Genes and Genomes (KEGG) database. The level 2 KEGG pathways were compared between the two periods based on *t-*test (*P* <0.05) and visualized in heatmap via the “pheatmap” package. Pearson correlation and linear regression between the differential KEGG pathways and main influencing factors were analyzed via the “corrplot” package.

All statistical analyses were performed in R environment (https://www.r-project.org/) unless otherwise mentioned.

## Results

### Phytoplankton Community and Environmental Parameters

A total of 52 species of phytoplankton belonging to seven phyla were identified across all samples during the study period. Among these species, there were 25 species of Chlorophyta, 9 species of Cyanophyta, 8 species of Bacillariophyta, 5 species of Euglenophyta, 2 species of Pyrrophyta, 2 species of Cryptophyta, and 1 species of Chrysophyta. Although surface cyanobacteria biomass decreased in July because of the heavy rainfall brought by a typhoon, severe cyanobacterial blooms were observed that begun in May and continued into November (Supplementary Figure 1). Colonial *Microcystis* spp. (mainly *Microcystis aeruginosa*) with an abundance over 10^7^ cells/L were the predominant species in the cyanobacterial bloom. Accordingly, March-April was determined as the non-cyanobacterial bloom (NCB) period and May-November as the cyanobacterial bloom (CB) period.

The environmental parameters showed clear temporal variations (Supplementary Table 1). WT gradually increased from March, peaked in August (29.3°C), and then decreased. The pH ranged from 6.23 to 8.51 throughout the sampling period, with the maximal value in August and the lowest value in October. DO and SD varied from 1.49 to 9.14 mg/L and from 0.17 to 0.71 m, respectively. In general, the variations of NH_4_-N, NO_2_-N, NO_3_-N, and TN all initially decreased and then increased, and their minimal value of 0.04, 0.02, 0.28, and 0.52 mg/L, respectively, was observed in June and July. The trends of phosphate (PO_4_-P) and total phosphorus (TP) were similar, with variations ranging from 0.05 to 0.15 mg/L and 0.06 to 0.83 mg/L, respectively.

### Composition of the Gut Microbial Community of *B. aeruginosa* in the Two Periods

**Figure 1:**
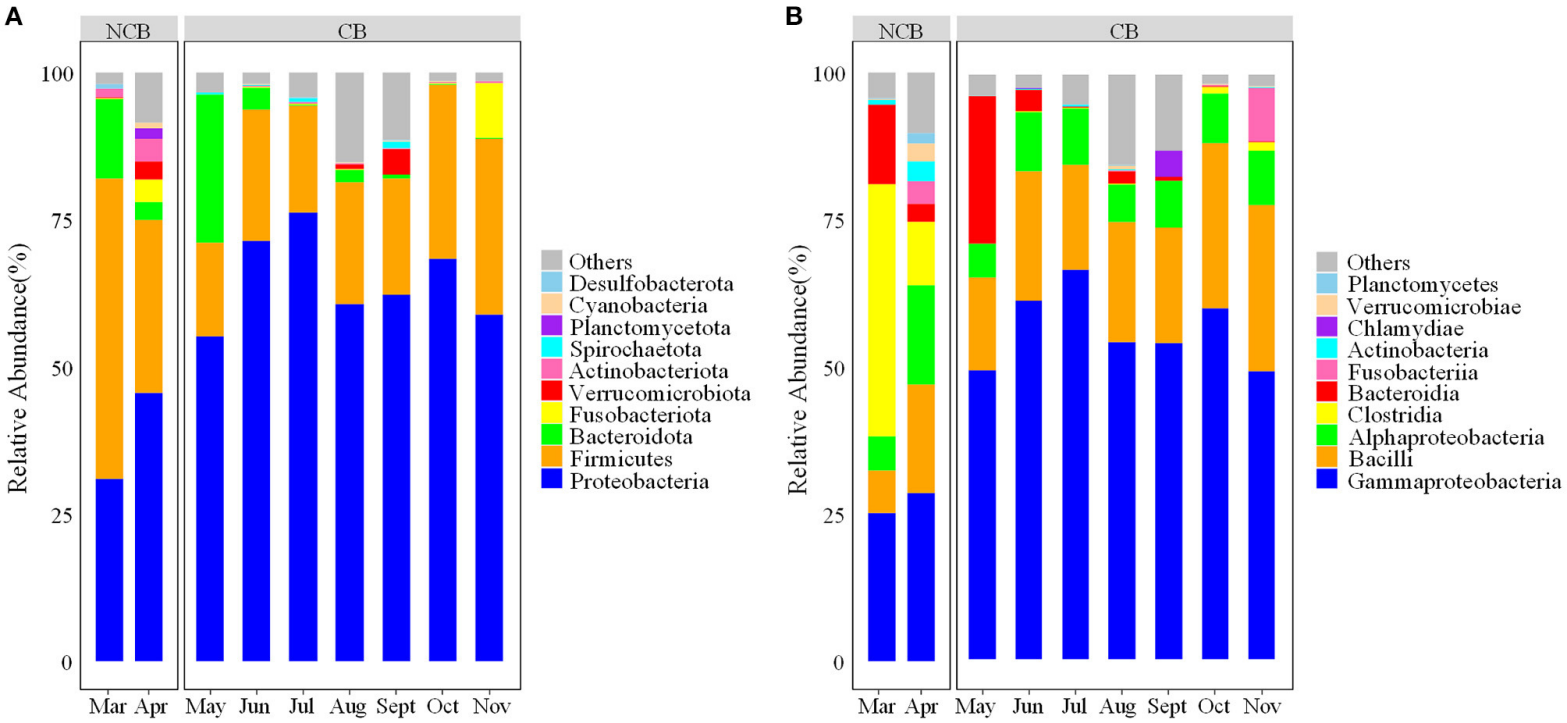
Composition of the bacterial community in the gut of *B. aeruginosa* snails **(A)** at the phylum level; **(B)** at the class level. NCB, non-cyanobacterial bloom; CB, cyanobacterial bloom.

A total of 2,489 ZOTUs belonging to 25 phyla and 311 genera were detected across the samples. Among these ZOTUs, 893 (35.9%) were shared by two distinct periods, 875 (35.2%) and 719 (28.9%) were unique in samples from the NCB and CB periods, respectively (Supplementary Figure 2). At the phylum level, Proteobacteria and Firmicutes were the two most dominant phyla in the gut of *B. aeruginosa* (Figure 1A), with an overall relative abundance of 58.8 and 26.3%, respectively. The relative abundance of Proteobacteria was lower during the NCB period but higher during the CB period. The opposite trend was observed in Firmicutes. Moreover, the relative abundance of Bacteroidota was higher during the NCB period than that during the CB period. At the class level, Gammaproteobacteria (49.8%) and Bacilli (19.9%) were the dominant classes, followed by Alphaproteobacteria (8.9%), Clostridia (6.3%), and Bacteroidia (5.4%). As shown in Figure 1B, the relative abundance of Gammaproteobacteria was lower but that of Clostridia was higher during the NCB period than those during the CB period.

**Figure 2:**
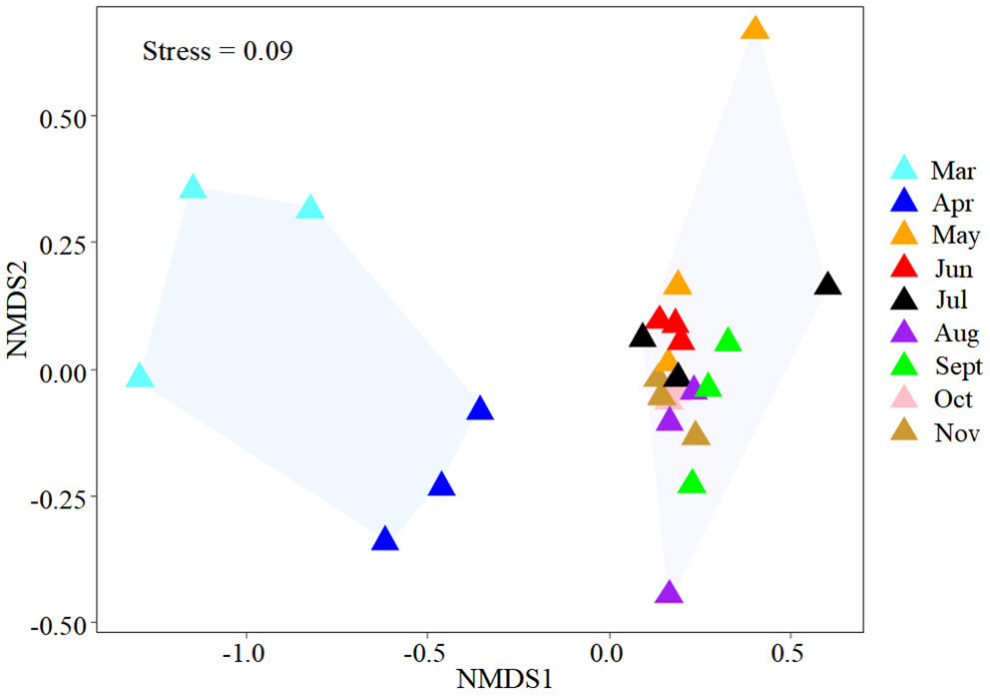
NMDS plot used to visualize the dissimilarities of the gut microbial communities of snails in different months.

**Figure 3:**
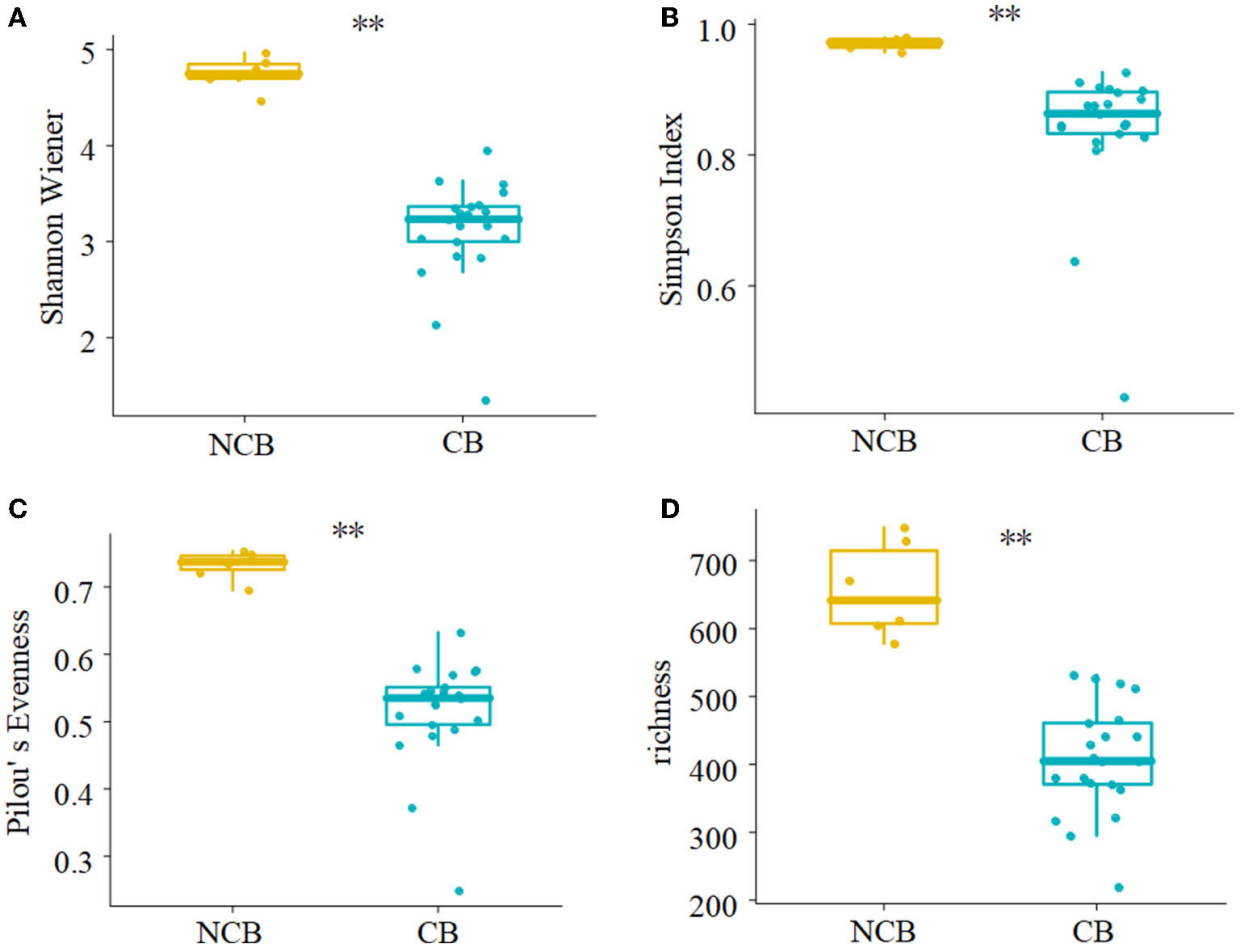
Comparison of alpha diversity indices of gut microbiota between non-cyanobacterial bloom (NCB) and cyanobacterial bloom (CB) periods. **(A)** Shannon-Wiener index, **(B)** Simpson index, **(C)** Pilou's evenness, **(D)** richness. **P < 0.01.

**Figure 4:**
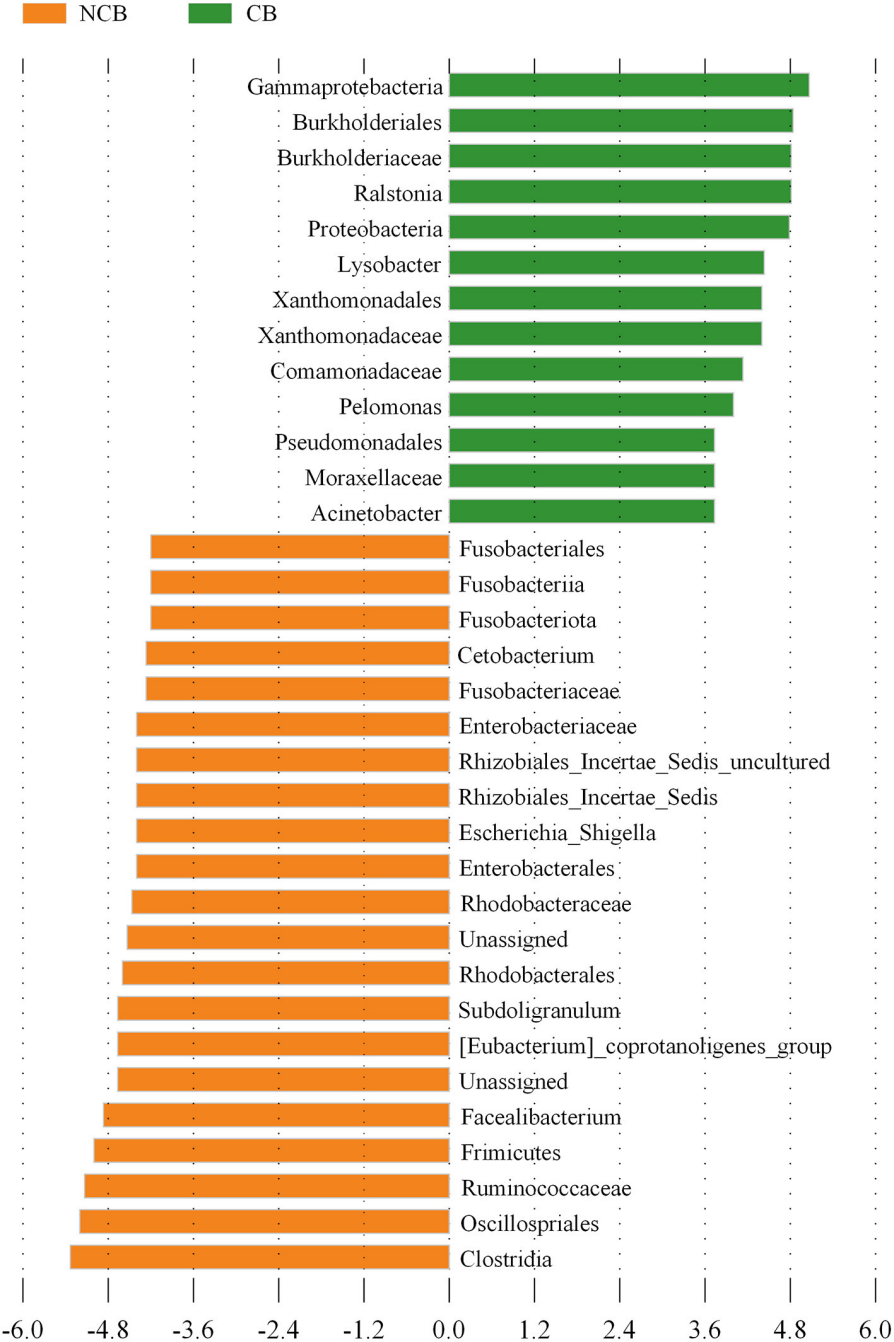
Bacterial taxa differentially represented between the non-cyanobacterial bloom (NCB) period and the cyanobacterial bloom (CB) period, as determined by LEfSe by using the default parameters.

NMDS was used to determine the similarity of the gut microbial community of *B. aeruginosa* in different months (Figure 2). The stress value was 0.09, indicating that the ordination pattern was acceptable and reliable. NMDS indicated that the snail samples clustered into two groups that corresponded to the NCB and CB periods. ANOSIM revealed significant differences between the two periods (*P* < 0.01, r = 0.884). The alpha diversity of the gut microbial community of *B. aeruginosa* was assessed. The four alpha diversity indices (Shannon-Wiener index, Simpson index, Pilou's evenness, and richness) were significantly higher during the NCB period than those during the CB period (*P* < 0.05), indicating that the bacterial richness and microbial diversity in the gut of *B. aeruginosa* decreased with the occurrence of cyanobacterial bloom (Figure 3). Microorganisms that were differentially represented in the two periods were identified by performing LEfSe on taxa with an LDA score > 2. Firmicutes (including the family [Eubacterium]_coprostanoligenes*_*group and the genus *Faecalibacterium, Subdoligranulum*), Fusobacteria (including the genus *Cetobacterium*), and Proteobacteria (including the family Rhodobacteraceae and the genus *Rhizobiales_Incertae_Sedis_uncultured, Escherichia-Shigella*) were more abundant during the NCB period than during the CB period (*P* < 0.05). Proteobacteria (including the genus *Ralstonia, Pelomonas, Acinetobacter*, and *Lysobacter*) were more abundant during the CB period than during the NCB period (Figure 4).

### Potential Relationships Between the Gut Microbial Community and Environmental Factors

**Figure 5:**
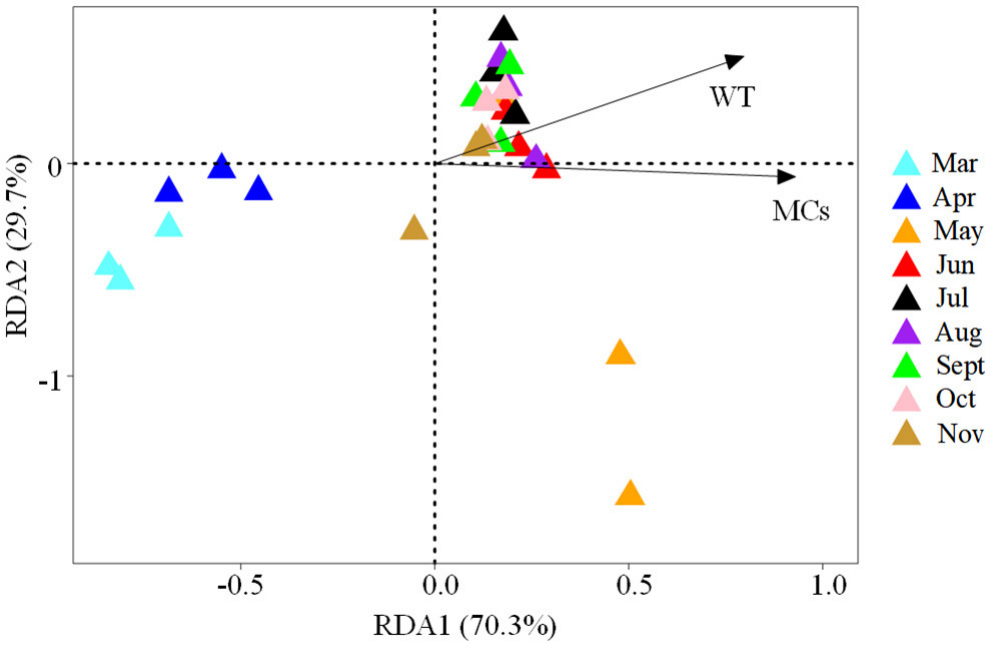
The biplot of the first two axes of the RDA analysis for the significant environmental factors associated with gut microbial community variation.

**Figure 6:**
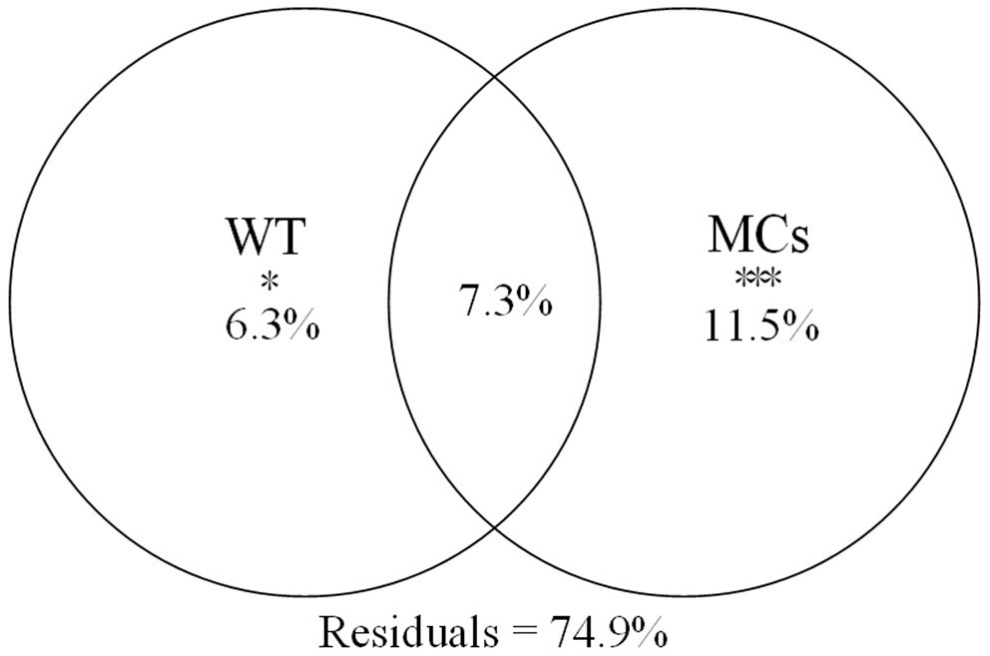
Variance partitioning plot of gut microbial community of *B. aeruginosa*. *P < 0.05, ***P < 0.001.

**Figure 7:**
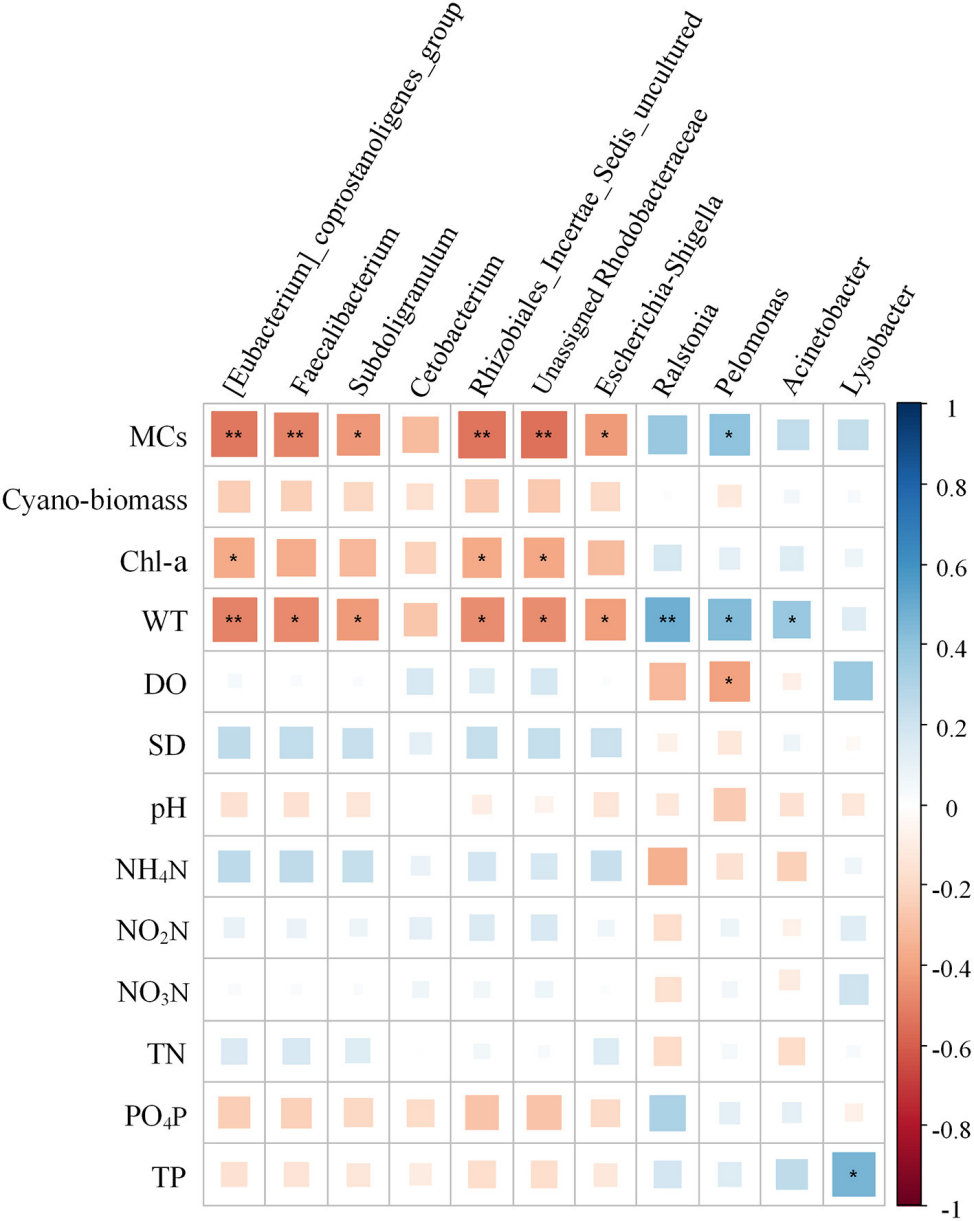
Relationship between the abundant discriminant taxa and environmental factors. The significance is noted as follows: **P < 0.01; *P < 0.05; and “blank,” not significant. Cyano-biomass: Cyanobacteria biomass.

Using the forward selection procedure, MCs (F = 4.508, *P* < 0.001) and WT (F = 1.989, *P* = 0.023) were selected as explanatory factors for gut microbial community variations. The relationship between gut microbial community and significant environmental factors was revealed by the RDA model. The first and second axes of the RDA model explained 70.3% and 29.7% of the variations, respectively (Figure 5). VPA revealed that the MCs alone effects explained 11.5% of the variations (*P* < 0.01), which was higher than that of the WT alone effects (6.3%, *P* < 0.05). The shared effects of MCs and WT factors explained 7.3% of the variations. The unexplained proportion was 74.9% (Figure 6). In addition, the abundant discriminant taxa of the two periods exhibited a certain correlation with the environmental variables. The abundant discriminant taxa during the NCB period exhibited negative correlations with MCs in tissues, WT, and Chl-*a*. The abundant discriminant taxa during the CB period were strongly correlated with WT, MCs, DO, and TP. For example, *Ralstonia* showed a positive correlation with WT, and *Lysobacter* displayed a positive correlation with TP (Figure 7).

### Prediction and Comparison of Gut Microbial Community Functions in the Two Periods

**Figure 8:**
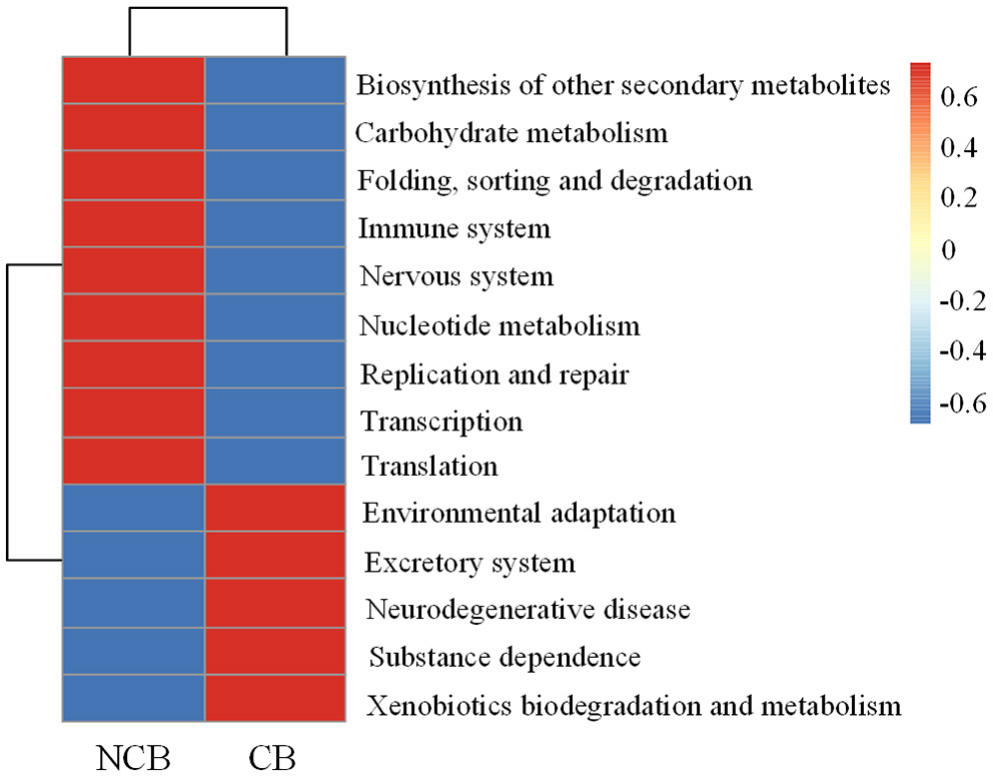
Differences in KEGG functions in the *B. aeruginosa* gut microbial community during non-cyanobacterial bloom (NCB) and cyanobacterial bloom (CB) periods (level 2). (*P* < 0.05).

**Figure 9:**
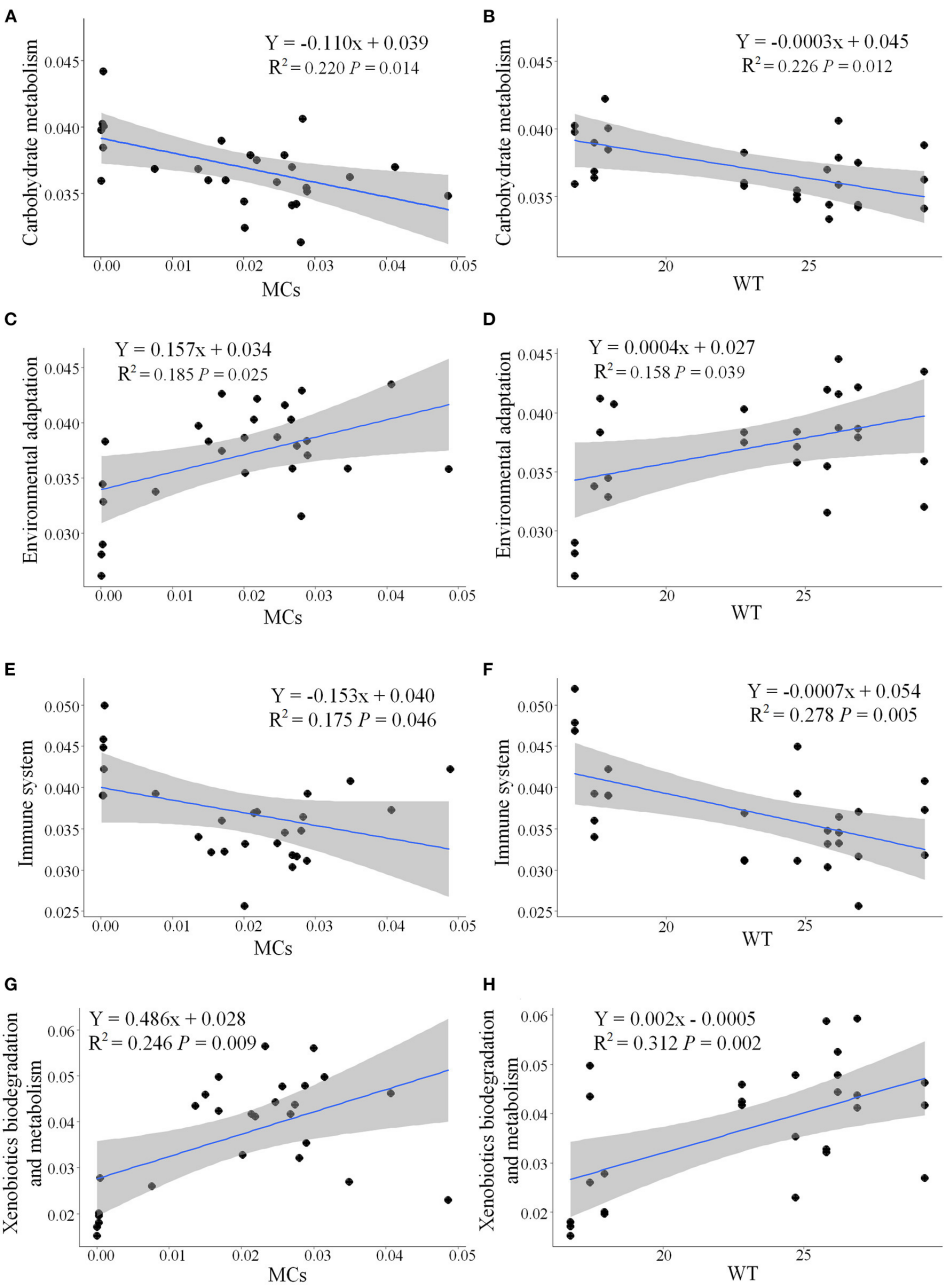
Linear relationships among the pathways carbohydrate metabolism **(A,B)**, environmental adaptation **(C,D)**, immune system **(E,F)**, and xenobiotics biodegradation and metabolism **(G,H)** and changes in MCs and WT. The R^2^ values represent correlation coefficient, *P*-values represent significant differences (*P* < 0.05). The gray-shaded area represents a 95% confidence interval.

Functional metabolic pathways of the gut microbial community 16S rRNA gene sequences were predicted by PICRUSt2. The mean Nearest Sequenced Taxon Index value was 0.10 ± 0.06. The level 1 KEGG pathway indicated a high abundance of genes related to metabolic pathways, environmental information processing, and genetic information processing (Supplementary Table 2). Nine pathways (e.g., carbohydrate metabolism and immune system) were more abundant during the NCB period, whereas five pathways (e.g., environmental adaptation and xenobiotics biodegradation and metabolism) were more abundant during the CB period (*P* < 0.05) (Figure 8). The results of significant regression analysis of the differential pathways and the two main influencing factors (MCs and WT) are shown in Figure 9. The pathways of carbohydrate metabolism, immune system, environmental adaptation, and xenobiotics biodegradation and metabolism showed a significant linear regression relationship with MCs and WT.

## Discussion

### Changes in and Influencing Factors of the Gut Microbiota Composition of *B. aeruginosa* Exposed to Cyanobacterial Bloom

The main gut microbial community composition of *B. aeruginosa* was similar to that of other aquatic snails, such as *Oncomelania hupensis* (Hao et al., 2020) and *Pomacea canaliculata* (Li et al., 2019). Proteobacteria, Firmicutes, and Bacteroidota constitute the prominent phyla in the intestines of freshwater gastropods. Although the gut microbiota compositions of different species of aquatic snails are relatively similar at the phylum level, notable differences may still be observed at other taxonomic levels. These differences are also common in the gut microbiota from different temporal (Hu et al., 2018) or spatial (Li et al., 2019) samples. In our case, the taxonomic composition and the alpha diversity of the gut microbiota of *B. aeruginosa* were variable between the NCB and CB periods (Figure 2). These results were consistent with previous field observations that the gut microbiota of aquatic snails could respond to different environmental conditions (Yang et al., 2019; Hao et al., 2020). Although no study has reported on the response of gut microbiota of gastropods to cyanobacterial blooms, the impacts of bloom-forming cyanobacteria and their toxins on intestinal flora have been confirmed in fish (Duperron et al., 2019; Qian et al., 2019) and cladocerans (Macke et al., 2017). The gut bacterial composition and diversity of aquatic gastropods can be determined by multiple factors, including environmental variation (Yang et al., 2019) and diet (Ito et al., 2019). Considering that these snails were all collected from the same habitat in the same location in this study, environmental fluctuations associated with blooms thus may have been the major driver that shaped the gut microbiota of *Bellamya*. Compared with other physicochemical factors of the habitat, MCs and WT were the main influencing factors on the gut microbial community of *B. aeruginosa*, and the contribution of MCs was greater than that of WT. This result suggested that the gut microbiome of *B. aeruginosa* can rapidly respond to changes in host environmental conditions, especially toxin accumulation in tissues, through changes in community composition.

Some indoor experiments have shown that temperature change determines the gut microbiota composition and function of aquatic animals (e.g., Li et al., 2018). However, unlike direct testing of the impacts of temperature under controlled experimental conditions, field studies cannot readily identify the associations between temperature and gut microbiota (Sepulveda and Moeller, 2020). Although multicollinearity among variables was eliminated through statistical approach in this study, and due to the fact that WT often covaries with other environmental variables (Sepulveda and Moeller, 2020), WT likely reflects the dynamics of unobserved or difficult-to-quantify variables, such as changes in food availability or sources. As a facultative suspension feeder, *B. aeruginosa* tend to obtain food by filter-feeding when sufficient food materials (such as edible algae) are present in the water column, and the presence of large concentrations of toxic cyanobacteria may result in a high percentage of food materials (such as detritus) being collected by scraping (Qiu et al., 2017). Since blooms usually occur during hot seasons, changes in food sources caused by the blooms frequently co-occurred with changes in water temperature. This may explain why the contribution of interaction between WT and MCs was also high, even higher than that of WT alone and the presence of such a high proportion of unexplained residuals.

### Responses of Potential Functions of Gut Microbiota of *B. aeruginosa* to Cyanobacterial Bloom

*Faecalibacterium* and *Subdoligranulum* (both belonging to the family Ruminococcaceae) are considered to be able to produce diverse organic acids and short chain fatty acids (Flint et al., 2012; Miquel et al., 2013), which help to enhance anti-inflammatory effects and improve the immunity of the hosts (Lopez-Siles et al., 2017). However, the relative abundance of these bacteria was higher during the NCB period than that during the CB period. Regression analysis revealed that the underlying metabolic functions related to immune system were negatively correlated with the increase of MCs content (Figure 9E), suggesting that the occurrence of cyanobacterial blooms weakened the host immunity mediated by the gut bacteria. Meanwhile, the decrease in carbohydrate metabolism related to MCs also reflected the inhibition of energy metabolism. However, the pathways related to environmental adaptation and xenobiotics biodegradation and metabolism were upregulated during the CB period, suggesting that the potential for host-microbial synergistic detoxification was increasing. Aquatic animals display similar responses to environmental stress, such as heavy metals (Yan et al., 2020) and chemical pollutants (Milan et al., 2019).

During cyanobacterial blooms, the major food source of *B. aeruginosa* changes to detritus (Qiu et al., 2017), which is believed to be mainly composed of allochthonous resources from terrestrial plants in small and shallow water bodies (Holgerson et al., 2016). Although terrigenous detritus usually consists of cellulose and lignin that most macroinvertebrates are unable to digest and assimilate, studies have found that the gut bacteria of freshwater snails can help their host degrade these indigestible substances (e.g., Brendelberger, 1997). We also found that the abundance of bacteria with similar functions substantially increased after the occurrence of cyanobacterial bloom. As plant pathogens, *Ralstonia* can secrete various plant cell wall-degrading enzymes (Poueymiro and Genin, 2009), and their increased abundance may help the snail host to adapt to changes in food source. Furthermore, some previous studies indicated that remarkable changes in the abundance of Rhodobacteraceae are associated with the switching of food or energy sources during metamorphosis in aquatic invertebrates (Zhang et al., 2020; Yang M. J. et al., 2022). In our results, the changes in these bacteria at different periods further reflected the response characteristics of the gut microbiota assembly of *B. aeruginosa* to food changes.

## Conclusion

Proteobacteria and Firmicutes were the prominent phyla of gut microbes of *B. aeruginosa*. The composition and relative abundance of the gut microbes were affected by environmental conditions, especially those related to toxic cyanobacteria. The alpha diversity of the gut microbiota of *B. aeruginosa* exposed to cyanobacterial bloom was lower than that in non-cyanobacterial bloom. The potential functions of the gut microbiome could help the snail meet its immunity and energy needs during cyanobacterial bloom stress. These results not only provide key information for revealing the response mechanism of freshwater snails to algal blooms but also helps clarify the biological adaptation mechanism of gastropod host from the perspective of intestinal flora.

## Data Availability Statement

The data presented in the study are deposited in the NCBI repository, accession number PRJNA822529.

## Author Contributions

TL: methodology, formal analysis, and writing – original draft. JZ: conceptualization, supervision, writing – review, editing, and funding acquisition. XY: investigation. WY: validation, writing – review, and editing. ZZ: writing – review and editing. All authors contributed to the article and approved the submitted version.

## Funding

This work was supported by National Natural Science Foundation of China (42077219), Hangzhou Municipal Agriculture and Social Development Project (2020ZDSJ0697), and Fundamental Research Funds for the Provincial Universities of Zhejiang (SJLY2020011).

## Conflict of Interest

The authors declare that the research was conducted in the absence of any commercial or financial relationships that could be construed as a potential conflict of interest.

## Publisher's Note

All claims expressed in this article are solely those of the authors and do not necessarily represent those of their affiliated organizations, or those of the publisher, the editors and the reviewers. Any product that may be evaluated in this article, or claim that may be made by its manufacturer, is not guaranteed or endorsed by the publisher.
