# Supplementary material for: Responses of Gut Microbial Community Composition and Function of the Freshwater Gastropod Bellamya aeruginosa to Cyanobacterial Bloom
Source: Front Microbiol. 2022 May 13;13:906278. doi: 10.3389/fmicb.2022.906278 (PMC9136413; doi:10.3389/fmicb.2022.906278)
Supplement: Supplementary file 1 [file Data_Sheet_1.docx]

**Supplementary Figures and Tables**

**
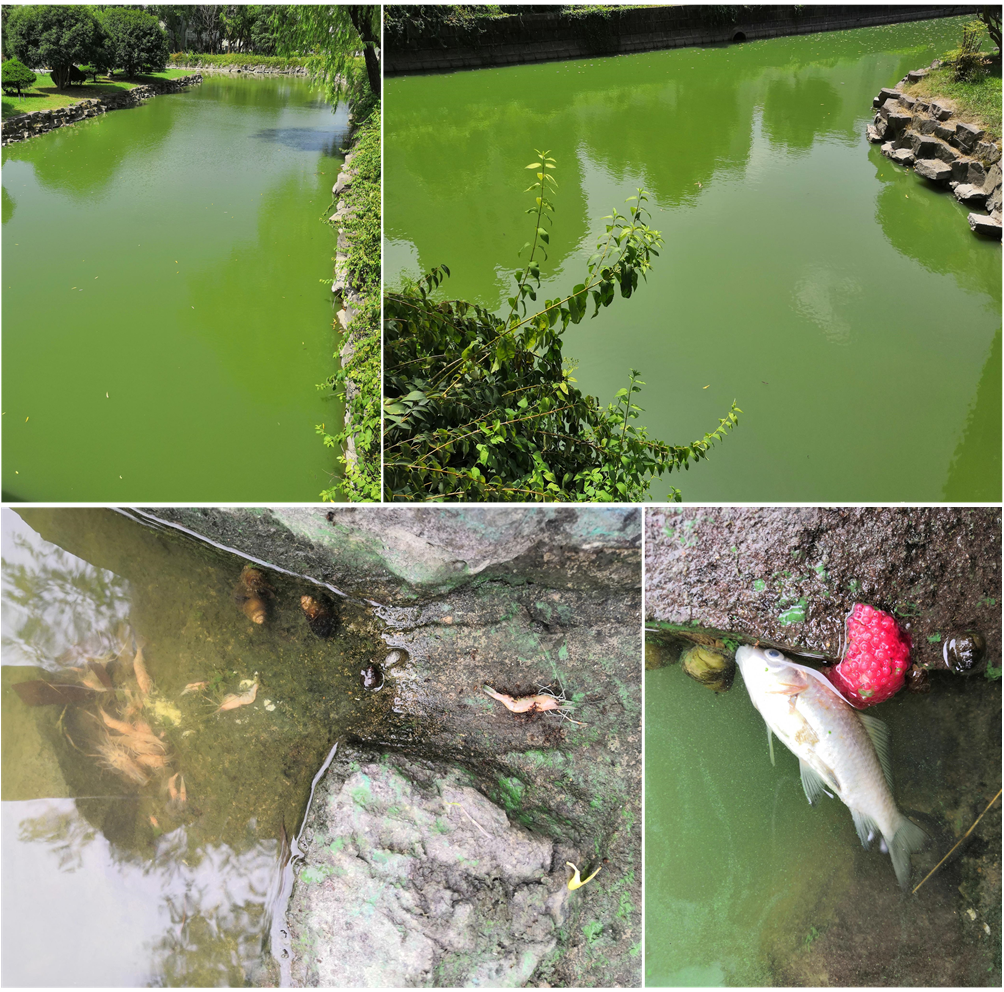
**

**Supplementary Figure 1.** Pictures of sampled water body during cyanobacterial blooms.


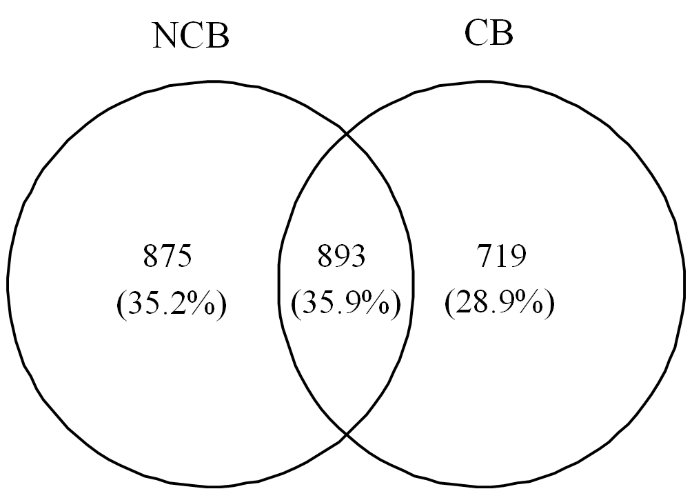


**Supplementary Figure 2.** Venn diagram showing shared and unique OTUs at 97% identity between the noncyanobacterial bloom (NCB) and cyanobacterial bloom (CB) periods.

**Supplementary Table 1.** Summary of environmental variables variation across sampling time (mean+SD).

|  | Mar. | Apr. | May. | Jun. | Jul. | Aug. | Sept. | Oct. | Nov. |
| --- | --- | --- | --- | --- | --- | --- | --- | --- | --- |
| WT (℃) | 16.7 | 17.9 | 24.7 | 26.2 | 26.9 | 29.3 | 25.8 | 22.8 | 17.4 |
| DO(mg/L) | 5.46±0.13 | 6.88±0.08 | 6.36±0.04 | 3.66±0.01 | 1.49±0.01 | 3.82±0.09 | 1.57±0.02 | 9.14±0.16 | 8.58±0.13 |
| SD(cm) | 61.00±0.82 | 58.00±0.82 | 38.90±0.49 | 71±0.82 | 60.00±1.63 | 17.4±0.22 | 27.00±2.45 | 56±0.82 | 46±0.82 |
| pH | 7.02±0.01 | 7.27±0.03 | 8.37±0.03 | 7.19±0.02 | 7.13±0.14 | 8.51±0.14 | 6.90±0.07 | 6.23±0.08 | 7.64±0.02 |
| NH_4_N(mg/L) | 0.27±0.01 | 0.22±0.01 | 0.22±0.03 | 0.07±0.01 | 0.04±0.01 | 0.10±0.01 | 0.30±0.01 | 0.26±0.01 | 0.28±0.04 |
| NO_2_N(mg/L) | 0.055±0.003 | 0.061±0.001 | 0.043±0.004 | 0.034±0.003 | 0.018±0.007 | 0.028±0.012 | 0.083±0.021 | 0.075±0.002 | 0.053±0.004 |
| NO_3_N(mg/L) | 0.689±0.009 | 0.720±0.002 | 0.521±0.008 | 0.307±0.003 | 0.278±0.007 | 0.614±0.029 | 1.037±0.091 | 0.890±0.008 | 0.898±0.031 |
| TN(mg/L) | 1.101±0.024 | 0.922±0.021 | 0.872±0.155 | 0.515±0.004 | 0.604±0.037 | 0.829±0.082 | 1.491±0.216 | 1.002±0.082 | 1.264±0.155 |
| PO_4_P(mg/L) | 0.060±0.008 | 0.055±0.002 | 0.099±0.008 | 0.085±0.003 | 0.121±0.006 | 0.145±0.010 | 0.058±0.004 | 0.071±0.013 | 0.049±0.003 |
| TP(mg/L) | 0.066±0.002 | 0.061±0.003 | 0.099±0.008 | 0.089±0.012 | 0.127±0.001 | 0.147±0.009 | 0.061±0.005 | 0.832±0.018 | 0.071±0.032 |
| Chl-*a*(µg/L) | 2.17±0.09 | 2.80±0.08 | 27.65±0.41 | 11.92±0.18 | 8.92±0.09 | 23.19±0.03 | 14.91±0.08 | 6.89±0.09 | 4.54±0.06 |

**Supplementary Table 2.** Relative abundance of the level 1 KEGG pathway.

| Level 1 | Relative abundance（%） |
| --- | --- |
| Metabolism | 77.24% |
| Human Diseases | 1.83% |
| Organismal Systems | 4.00% |
| Environmental Information Processing | 6.34% |
| Cellular Processes | 4.96% |
| Genetic Information Processing | 5.62% |
